# Editorial: Plant cuticle: From biosynthesis to ecological functions

**DOI:** 10.3389/fpls.2023.1154255

**Published:** 2023-02-15

**Authors:** Isabel Molina, Amauri Bueno, Antonio Heredia, Eva Domínguez

**Affiliations:** ^1^ Department of Biology, School of Life Sciences and the Environment, Algoma University, Sault Ste Marie, ON, Canada; ^2^ Chair of Botany II – Ecophysiology and Vegetation Ecology, Julius von Sachs Institute of Biological Sciences, University of Würzburg, Würzburg, Germany; ^3^ Departamento de Biología Molecular y Bioquímica, Instituto de Hortofruticultura Subtropical y Mediterránea “La Mayora”, Universidad de Málaga-Consejo Superior de Investigaciones Científicas, Universidad de Málaga, Málaga, Spain; ^4^ Departamento de Mejora Genética y Biotecnología, Instituto de Hortofruticultura Subtropical y Mediterránea “La Mayora”, Universidad de Málaga-Consejo Superior de Investigaciones Científicas, Estación Experimental La Mayora, Málaga, Spain

**Keywords:** plant cuticle, waxes, cutin, cell wall, phenolics, water transpiration, pathogens

At the interface between the plant and the environment, the cuticle functions as a barrier to water loss and as a protective layer against pathogens and UV light (Kunst and Samuels, 2009; Yeats and Rose, 2013). Over the last decades, numerous researchers from different fields have investigated the chemistry, structure, biosynthesis and functional properties of the plant cuticle, bringing new questions and insights, and significantly enriching our understanding of its structure, chemical composition, physical properties, natural variability, development, and functions.

In the current scenario of global climate change, it is crucial to understand the biophysical properties of the cuticle as well as how environmental factors, including temperature, UV radiation and relative humidity, modulate cuticle deposition and its functional properties (Domínguez et al., 2011). Seufert et al. report on the influence of different wax fractions on the water transpiration properties of isolated leaf cuticles. Using a differential extraction protocol, the authors studied the influence of triterpenoids and very-long-chain aliphatics (VLCA) on water permeability in cuticles from several species. Comparison of water permeance from isolated cuticles and selectively dewaxed cuticles showed that the cuticle components that greatly affect its transpiration barrier function are mainly VLCAs, and that triterpenoids barely contribute to the water barrier properties of the cuticle. Chen et al. compare adaxial and abaxial cuticle transpiration in detached tea leaves from eight different cultivars. Total leaf cuticle transpiration correlated with abaxial transpiration. Among the different wax classes, VLCA and glycol esters negatively correlated with leaf cuticle transpiration. Additionally, intracuticular waxes were the major water barrier in the adaxial leaf surface and epicuticular waxes in the abaxial side. Low water transpiration rate is also important for extending the life of cut ornamental flowers. Cheng et al. used oriental lily to investigate flower cuticle transpiration and wax chemical composition, showing a higher amount of waxes, largely *n*-alkanes, in tepals than in leaves. However, leaves presented lower water permeance than tepals, a result that the authors attributed to differences in alkane chain length, with C27 and C29 predominating in tepals and C29 and C31 being more prevalent in leaves. Exploration of these results in other species could provide a tool to select for cultivars with extended vase life.

The Michaelis´ hypothesis states that inadequate cuticle development can cause increased transpiration rate in the treeline. Bueno et al. examine this hypothesis in populations of *Pinus uncinata* from the subalpine forest and the alpine tundra. The authors found higher minimum conductance, lower amounts of cuticle waxes and thinner cuticles in needles from the tundra trees than in those from the forest. These results support the notion that inadequate cuticle development could be one of the factors leading to increased water transpiration during winter in this species. Vega et al. describe water sorption and desorption of isolated leaf cuticles of three woody species. The presence of cutan in the cuticles of holly and cherry laurel was associated with higher water sorption. Differences in cuticle water sorption and water loss were detected among the three studied species, whereas an effect of leaf age on water desorption was only observed in eucalyptus.

The mechanical, thermal and optical properties of the tomato fruit cuticle were analyzed by Benítez et al. and Benitez et al. Cutinization of anticlinal cell walls during development plays a biomechanical role inducing cuticle softening. The increase in cuticle phenolic content with ripening was accompanied by a progressive stiffness that suggested a cooperative association of phenolics with both cutin and polysaccharide fractions. The heat regulation capacity of the cuticle varied with development and temperature and was mainly attributed to the cutin matrix. However, the glass transition temperature, the transition from a rigid glassy state to a relaxed rubbery conformation, was modulated by phenolics, polysaccharides and, to a minor extent, waxes. The observed increase in cuticle glass transition between fruit growth and ripening implies physical changes that can affect molecular diffusion through the cuticle. The authors also report that the highly efficient UV-B filtering capacity of the cuticle can be attributed to the accumulation of cinnamic acids, and that the deposition of the flavonoid chalconaringenin during ripening expands the blocking capacity of the cuticle to include UV-A light.

Tomato fruit has become a model for cuticle analysis due to its thick and easy-to-isolate cuticle, as well as to the genetic and genomic resources available in the form of natural and artificially induced mutants, germplasm collections, and intra and interspecies segregating populations (Petit et al., 2017). In their minireview, Petit et al. explore the recent advances in uncovering the genetic and molecular determinants of cuticle deposition and its implications in our understanding of tomato fruit cuticle and its relation to plant growth and performance. Future challenges and possible avenues of research such as investigating the regulation of cuticle phenolics and cell wall polysaccharides deposition, the correlation of cuticle deposition with epidermal cell development and organ growth, and the epigenetic regulation of cuticle biosynthesis are also considered.

Over the last years, there has been a shift in how the cuticle is envisioned, from an isolated outer layer to part of the more complex scenario of the outer epidermal cell wall (Ingram and Nawrath, 2017). In a mini-review, Reynoud et al. describe recent developments and hypotheses on how cuticle chemical composition can affect its architecture and the architecture-function relationships. Although plant cuticles have been typically described as an assembly of lipids, namely waxes and cutin, a far more complex architecture of the cuticle is depicted. It can be described as a hydrophobic, dynamic, chemically and spatially heterogeneous composite containing lipids, cell-wall-derived polysaccharides, phenolic acids and, in some instances such as ripe tomato, flavonoids. The relationship between cell wall pectin, cuticle permeability and *Botrytis cinerea* resistance was investigated by Lorrai et al. using *Arabidopsis thaliana* pectin mutants. Alteration of homogalacturonan integrity, a major pectin component, increased the accumulation of reactive oxygen species (ROS) dependent on the activity of the class III peroxidase AtPRX71. These changes in homogalacturonan structure also led to a notable increase in cuticle permeability and resistance to *B. cinerea*. Interestingly, neither cuticle permeability nor pathogen resistance were affected in cell wall mutants displaying changes in other polysaccharide components. In parallel, Aragon et al. report on the contribution of cutin and waxes to cuticle permeability, ROS accumulation and sensitivity to *B. cinerea* in *A. thaliana* mutants. Although mutants with altered cutin or wax composition displayed an increase in cuticle permeability and ROS levels, resistance to *B. cinerea* was only found in cutin mutants. These mutants displayed upregulation of genes related to pectin and ROS accumulation after *B. cinerea* inoculation. These observations point again to a relationship between pathogen resistance, the pectin domain of the epidermal cell wall, cuticle permeability and cutin composition that merits further study. Arya et al. review the complex and multidimensional interactions between the plant cuticle and pathogenic fungi, paying special attention to epicuticular waxes and cutin monomers, the two most studied cuticle components in relation to pathogens. Differences between pre-penetration and infection processes, fungi lifestyles, and epicuticular wax chemistry and structure make this topic particularly challenging. The authors further debate how composition, structure, permeability and released cutin monomers are perceived by plants and can elicit defense responses.

Ensuring peak fresh produce quality is one of the main current goals of the agri-food industry (Oltra-Mestre et al., 2021). The biophysical properties of the cuticle modulate traits related to postharvest life (Fernández-Muñoz et al., 2022). Si et al. study the radial growth of apple cuticle and its implications in mechanical strain build-up, a trigger for several fruit surface disorders. A combination of radioactive labelling and cuticle analysis was employed showing that cuticle deposition occurred in the inner cuticle region, close to the cell wall, thus creating an aging gradient across cuticle thickness. The authors suggest that this pattern of cuticle deposition could delay surface microcrack propagation to epidermal cells in species with continuous cuticle deposition throughout development. Hurtado and Knoche investigate the role of the cuticle in strawberry water soaking, a skin disorder that limits open field production. Moisture exposed areas were associated with the presence of cuticle microcracks, which acted as fast and localized water uptake areas, thus triggering a series of events leading to cell burst and cell content leakage. The relationship between cuticle chemical composition and postharvest fruit quality was investigated in zucchini and wampee. Carvajal et al. compare cuticle waxes and transcriptomic changes in a cold-tolerant and a cold-sensitive variety of zucchini at harvest and after cold storage. A thicker cuticle and higher amount of waxes were found in the cold-tolerant variety. The authors conclude that in zucchini, cuticle thickness is related to chilling tolerance and helps to reduce water loss, highlighting the importance of the biosynthesis of very-long-chain alkanes and its transcriptional regulation during the adaptation of the zucchini fruit to low temperatures and in maintaining the postharvest quality of zucchini fruit during cold storage. Huang et al. report on wampee cuticle composition and fruit transpiration along ripening in two cultivars. The cutin matrix was chiefly composed of dihydroxy fatty acids and the main wax compounds were alkanes and triterpenoids. Cultivar differences and changes during ripening were observed for the amount of cuticle and waxes. Fruit transpiration decreased with ripening. A negative correlation was found between the water barrier properties and the alkane content of ripening wampee fruits.

Two contributions to this Research Topic focus on largely unexplored areas of plant cuticle research. Philipe et al. review known aspects and hypothetical scenarios of the mechanisms used for the transport of cutin precursors and waxes across the plasma membrane and through the cell wall. The authors summarize the well-documented mechanism of cutin precursor export through the plasma membrane-localized ATP-binding cassette transporters. The researchers also discuss more exploratory and unknown aspects of the cellular trafficking and export of cuticle components *via* vesicles and *via* a non-vesicular route, and how the cuticle precursors can traverse the polysaccharide cell wall before their incorporation into the cuticle. The method article by Bock et al. is focused on the use of Raman vibrational microscopy, a non-destructive and fast technique that can be employed in epidermal tissue samples, to elucidate the in-depth localization and distribution of cuticle components at the microscale. After application of the methodology to study cuticles from three species, the authors reveal common cuticle chemical distribution features as well as differences, and draw general conclusions on the localization of waxes, phenolics and cutin on a supramolecular polysaccharide matrix.

The original article by Liu et al. describes the functional characterization of a ubiquitin ligase named ABA-related RING-type E3 ligase (AtARRE) that negatively regulates cuticle wax biosynthesis in *A. thaliana*. Co-expression of AtARRE and candidate target proteins involved in alkane formation showed that the alkane biosynthetic enzymes CER1 and CER3 are targets of AtARRE and that CER1 can be ubiquitinated by AtARRE. The authors propose that AtARRE serves as a post-translational regulator that terminates wax biosynthesis *via* the alkane-forming pathway.

Many uncertainties remain regarding the biogenesis and functions of the plant cuticle. To gain a broader perspective on the role of the cuticle in plant performance, it will be crucial to understand the structural and functional properties of isolated cuticles in their natural scenario, and to also consider cuticle interactions with epidermal cells. Because of its heterogeneous and composite nature, the cuticle varies among species and is modified by environmental, developmental and hormonal cues (Yeats and Rose, 2013). Hence, a comprehensive analysis of the interactions among cuticle components, their involvement in the functions ascribed to the cuticle, and how such components change in response to internal and external signals is required. In this sense, the design of artificial cuticles could be a promising strategy to study the contributions of individual cuticle components to the supramolecular structure and properties of the cuticle.

## Author contributions

All authors listed have made a substantial, direct, and intellectual contribution to the work and approved it for publication.
